# The α-Hemolysin nanopore transduction detector – single-molecule binding studies and immunological screening of antibodies and aptamers

**DOI:** 10.1186/1471-2105-8-S7-S9

**Published:** 2007-11-01

**Authors:** Stephen Winters-Hilt

**Affiliations:** 1 Research Institute for Children, Children's Hospital, 200 Henry Clay Ave., New Orleans, LA 70118, USA; 2 Department of Computer Science, University of New Orleans, New Orleans, LA, 70148, USA

## Abstract

**Background:**

Nanopore detection is based on observations of the ionic current threading a single, highly stable, nanometer-scale channel. The dimensions are such that small biomolecules and biopolymers (like DNA and peptides) can translocate or be captured in the channel. The identities of translocating or captured molecules can often be discerned, one from another, based on their channel blockade "signatures". There is a self-limiting aspect to a *translocation*-based detection mechanism: as the channel fits tighter around the translocating molecule the dynamic range of the ionic current signal is reduced. In this study, a lengthy, highly structure, high dynamic-range, molecular *capture *is sought as a key component of a *transduction*-based nanopore detection platform.

**Results:**

A specialized role, or device augmentation, involving bifunctional molecules has been explored. The bifunctional molecule has one function to enter and blockade the channel in an information-rich self-modulating manner, while the other function is for binding (usually), located on a non-channel-captured portion of the molecule. Part of the bifunctional molecule is, thus, external to the channel and is free to bind or rigidly link to a larger molecule of interest. What results is an event transduction detector: molecular events are directly transduced into discernible changes in the stationary statistics of the bifunctional molecule's channel blockade. Several results are presented of nanopore-based event-transduction detection.

**Conclusion:**

It may be possible to directly track the bound versus unbound state of a huge variety of molecules using nanopore transduction detection.

## Introduction

Channel current based nanopore-transduction cheminformatics provides a new, incredibly versatile, method for transducing single molecule events into discernable channel current blockade signals. These discernible blockade patterns or statistics (i.e., stationary statistics regions) are hypothesized to correlate with molecular states, such as binding states or conformational states.

Sophisticated machine learning software has been brought to bear on this type of signal analysis. These software tools are web accessible [1], and have also been optimized for speed and integrated into the nanopore detector for "real-time" pattern-recognition informed (PRI) feedback [1]. Additional methods have been developed for distributed HMM and SVM (standard chunking [2]) to enable the processing speedup needed to perform real-time PRI-feedback.

A study of an antibody with linkage to a dsDNA molecule at its carboxy terminus is described. The dsDNA component is designed to be an excellent channel blockade modulator. The antibody component is designed to bind biotin. A simpler, direct analysis where the antibody is both blockade modulator and binding molecule is described in [3]. Similar studies of TF/TFBS (TBP binding to TATA box) are also performed [4]. Other studies of antibody- and aptamer-based biosensing and immunological screening protocols are being developed [5]. The prospects for single molecule biophysics and biochemistry, directed molecular design, and rapid immunological screening look very promising with use of channel current transduction detection.

The Background for nanopore blockade detection is given first, then the augmentation is described to make a nanopore *transduction *detector – a molecular "wrench" is quite literally thrown into the works. The rest of the Background introduces preliminary nanopore-based event transduction efforts, to be directly followed by the Results section with the latest results on nanopore transduction detection and the latest machine learning based software developments and results in managing the associated data analysis.

## Background

### The alpha-Hemolysin nanopore blockade detector

Single biomolecules, and the ends of biopolymers such as DNA, have been examined in solution with nanometer-scale precision using nanopore blockade detection [6-11]. In early studies [11], it was found that complete base-pair dissociations of dsDNA to ssDNA, "melting", could be observed for sufficiently short DNA hairpins. In later work [8,10], the nanopore detector attained Angstrom resolution and was used to "read" the ends of dsDNA molecules, and was operated as a chemical biosensor. In [6,7,9], the nanopore detector was used to observe the conformational kinetics of the end regions of individual DNA hairpins.

The notion of using channels as detection devices dates back to the Coulter counter [12], where pulses in channel flow were measured in order to count bacterial cells. Cell transport through the Coulter counter is driven by hydrostatic pressure – and interactions between the cells and the walls of the channel are ignored. Since its original formulation, channel sizes have reduced from millimeter scale to nanometer scale, and the detection mechanism has shifted from measurements of hydrostatically driven fluid flow to measurements of electrophoretically driven ion flow. Analytes observed via channel measurements are likewise reduced in scale, and are now at the scale of single biomolecules such as DNA and polypeptides. For nanoscopic channels, interactions between channel wall and translocating biomolecules can't, usually, be ignored. On the one hand this complicates analysis of channel blockade signals immensely, on the other hand, tell-tale on-off kinetics are revealed for binding between analyte and channel, and this is what has allowed the probing of intramolecular structure on single DNA molecules [6-10].

Biophysicists and medical researchers have performed measurements of ion flow through *single *nanopores since the 1970's [13]. The use of very large (biological) pores as polymer sensors is a relatively new possibility that dates from the pioneering experiments of Bezrukov et al. [14,15]. Their work proved that resistive pulse measurements, familiar from cell counting with the Coulter counter, could be reduced to the molecular scale and applied to polymers in solution. A seminal paper, by Kasianowicz et al., 1996 [16], then showed that *individual *DNA and RNA polymers could be detected via their translocation blockade of a nanoscale pore formed by α-hemolysin toxin. In such prior nanopore detection work, the data analysis problems were also of a familiar "Coulter event" form – where the event was associated with a current blockade at a certain, fixed, level. A more informative setting is possible with nanometer scale channels, however, due to non-negligible interaction between analyte and channel. In this situation the blockading molecule might not necessarily provide a *single*, fixed, current reduction in the channel, but will modulate the ion flow through the channel by imprinting its binding interactions (with the channel) and conformational kinetics on the confined channel flow environment. This is a very brief and limited synopsis of the Nanopore Detector background relevant to this paper. For other references on Nanopore Detectors use is made of a Nanopore Detector review presented in [17]: early work involving alpha-Hemolysin Nanopore Detectors can be found in [8-11,16-25]; rapidly growing research endeavors on Nanopore Detectors based on solid-state, and other synthetic, platforms can be found in [26-36].

**Figure 1:**
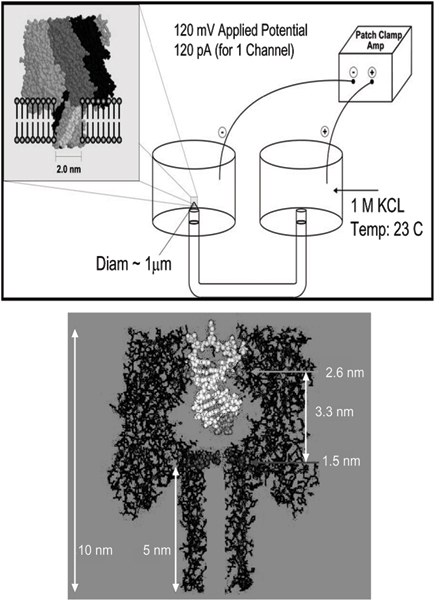
**The alpha-Hemolysin nanopore blockade detector with a 9 bp DNA hairpin shown to-scale in the *cis*-vestibule**. The upper panel shows the electrochemistry setup for the nanopore device. The lower panel shows the crystallographic description of the α-hemolysin channel with a nine base pair DNA hairpin superimposed.

The α-hemolysin (α-HL) channel, a protein heptamer formed by seven identical 33 kD protein molecules secreted by *Staphylococcus aureus*, is used as the channel in the nanopore device due to its stable conformation (in the strongly favored heptamer formation, which has minimal gating) and its overall geometry (see Fig. 1): the total channel length is 10 nm and is comprised of a 5 nm *trans*-membrane domain and a 5 nm vestibule that protrudes into the aqueous *cis *compartment [37]. The narrowest segment of the pore is a 1.5 nm-diameter aperture [37]. By comparison, a single strand of DNA is about 1.3 nm in diameter. Given that water molecules are 0.15 nm in diameter, this means that one hydration layer separates ssDNA from the amino acids in the limiting aperture. This places the charged phosphodiester backbone, hydrogen bond donors and acceptors, and apolar rings of the DNA bases within one Debye length (3 Å in 1 M KCl) of the pore wall (see Fig. 1). Not surprisingly, DNA and RNA interaction with the α-hemolysin channel during translocation is non-negligible (but not too strong either, i.e., it is not such that the molecule "gets stuck"). Although dsDNA is too large to translocate, about ten base-pairs at one end can still be drawn into the large cis-side vestibule. This permits very sensitive experiments since the ends of "captured" dsDNA molecules can be observed for extensive periods of time to resolve features, allowing highly accurate classification of the captured end of dsDNA molecules [6-10].

Channel current feature extraction methods, using Hidden Markov Models (HMMs) have also been designed for tracking individual-molecule *conformational *changes on the ends of captured biomolecules (biopolymers) [6,7,9]. The HMM tools were used to help systematically explore DNA dinucleotide flexibility, with particular focus on HIV's highly conserved (and highly flexible/reactive) viral DNA terminus. One of the most critical stages in HIV's attack is the binding between it's retroviral DNA and the retroviral integrase, which is influenced by the dynamic-coupling induced high flexibility of a CA/TG dinucleotide positioned precisely two base-pairs from the blunt terminus of the duplex viral DNA. The observed state kinetics of the DNA hairpins containing the CA/TG dinucleotide provides clear evidence for HIV's selection of a peculiarly flexible/interactive DNA terminus [38,4].

**Figure 2:**
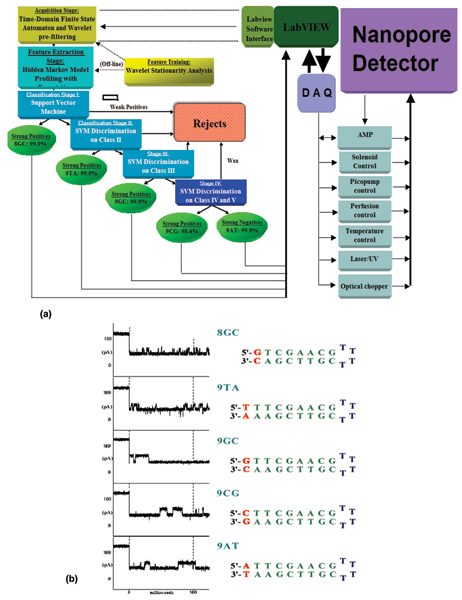
**(a) Nanopore cheminformatics & control architecture**. The figure shows the signal processing architecture that was used to classify DNA hairpins with this approach: Signal acquisition was performed using a time-domain, thresholding, Finite State Automaton. Hidden Markov Model processing with Expectation-Maximization was used for feature extraction on acquired channel blockades. Classification was then done by Support Vector Machine – the architecture for resolving the five DNA hairpin controls is shown. Four DNA hairpin control molecules have nine base-pair stem lengths that only differed in their blunt-ended DNA termini, the fifth control was an eight base-pair DNA hairpin. The accuracy shown is obtained upon completing the 15^th ^single molecule sampling/classification (in approx. 6 seconds), where SVM-based rejection on noisy signals was employed. In recent augmentations to this architecture, a LabWindows Server is now used. Data is then sent to cluster of Linux Clients via TCP/IP channel. Linux clients run expensive HMM analysis as distributed processes (similarly for off-line SVM training). The sample classification is used by the Server to provide feedback to the nanopore apparatus to increase the effective sampling time on the molecules of interest (this can boost nanopore detector productivity by magnitudes). A test case of such sampling-control feedback is shown in [1]. **(b) DNA hairpin controls and their diagnostic signals. **The secondary structure of the DNA hairpins studied is shown on the right, with their highest scoring diagnostic signals shown on the left. Each signal trace start at approximately 120 pA open channel current and all blockade in a range 40–60 pA upon "capture" of the associated DNA hairpin. Even so, the signal traces have discernibly different blockade structure, which can be extracted using a Hidden Markov Model (see [8] for further details).

Fig. 2 shows the pattern recognition informed signal processing architecture [8], with sampling feedback control [1]. The processing is designed to rapidly extract useful information from noisy blockade signals using feature extraction protocols, wavelet analysis, Hidden Markov Models (HMMs) and Support Vector Machines (SVMs). For blockade signal acquisition and simple, time-domain, feature-extraction, a Finite State Automaton (FSA) approach is used [39] that is based on tuning a variety of threshold parameters. A generic HMM is then used to characterize current blockades by identifying a sequence of sub-blockades as a sequence of state emissions [6-9,11]. The parameters of the generic-HMM can then be estimated using a method called Expectation/Maximization, or 'EM" [40], to effect de-noising. The HMM method with EM is part of the standard implementation used in what follows. Classification of feature vectors obtained by the HMM for each individual blockade event is then done using SVMs. For the nanopore detector augmented with auxiliary molecules much more data is usually needed to properly train the Machine Learning algorithms. The distributed training of these algorithms (recently established in [2]) is a critical component in real-time signal processing [1].

### The alpha-Hemolysin nanopore transduction detector

**Figure 3:**
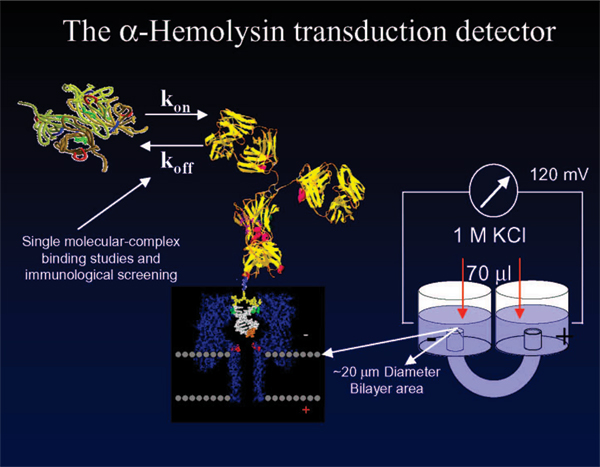
**The alpha-Hemolysin nanopore transduction detector**. The nanopore-based transduction detector uses a reporter molecule that binds to certain molecules, with subsequent distinctive blockade by the bound-molecule complex. An example of this is shown – the DNA-hairpin/Antibody complex. The interaction of that antibody with its target antigen is expected to lead to a blockade shift upon antigen binding, as shown in the Results. The latter description provides the general mechanism for directly observing the *single-molecule *antigen-binding affinities of any antibody in complex situations, such as those involving selection of adjuvants.

The improved detector sensitivity with toggling-type auxiliary molecules, or with bifunctional molecules, opens the door to a new, highly precise, means for examining the binding affinities between any two molecules, all while still in solution. The bifunctional molecules that have been studied on the nanopore detector include antibodies and aptamers [3-5], where the linked-to-modulator method is used (detailed in Methods) to track states on an individual molecular binding event (see Fig. 3). The molecular binding events studied were chosen to also demonstrate the specific utility of this device in drug candidate screening. Antibodies that bind strongly to target antigen can be good, same for aptamers in many situations. Sometimes a weak-binding is desired, when the drug is a toxin, for example, where the strategy might be to deliver the toxin with a weak-binding agent such that the toxin may eventually be cleared. Antibody-antigen interaction strength with different adjuvant types/amounts can be directly examined. Likewise, antibody-antigen states can be studied insofar as the binding at their effector region by introducing T-cell receptor binding sites (see Discussion).

There are important distinctions in how a nanopore detector can function: direct vs. indirect measurement of the signal. The channel blockade signals can be static, stationary, semi-stationary (phases with different stationary statistics), or non-stationary. A nanopore-based detector can *directly *measure molecular characteristics in terms of the blockade properties of individual molecules – this is possible due to the kinetic information that is embedded in the blockade measurements, where the adsorption-desorption history of the molecule to the surrounding channel, and the configurational changes in the molecule itself directly, imprint on the ionic flow through the channel [6-11]. This approach offers prospects for DNA sequencing and single nucleotide polymorphism (SNP) analysis [11].

The nanopore-based detector works *indirectly *if it uses a reporter molecule that binds to certain molecules, with subsequent distinctive blockade by the bound-molecule complex. One example of this, with the established DNA experimental protocols, is exploration of transcription factor binding sites via the different dsDNA blockade signals that occur with and without DNA binding by a hypothesized transcription factor. Similarly, a channel-captured dsDNA "gauge" that is already bound to an antibody could provide a similar blockade shift upon antigen binding to its exposed antibody as shown in the Results. The latter description provides the general mechanism for directly observing the *single molecule *antigen-binding affinities of any antibody.

The modulatory auxiliary molecule represents a new "wrench in the works", a wrench that happens to rattle around in a useful fashion, creates a new, much more sensitive, overall mechanism – one where it is possible to ***transduce ***single molecule events (such as intermolecular binding, intramolecular conformational change, and conformationally-mediated binding) into changes in the ionic current *stationary statistics*. More than an on/off event detection, the transduction is analog, with a range of values, such as binding strength available from binding event dwell time distributions (where event dwell times correspond exactly with the dwell time in an associated phase of ionic current stationary statistics).

The "wrench" that rattles in the channel (the auxiliary molecule) is chosen to provide highly modulatory blockades that change the most discernibly upon target binding. There is thus a molecular engineering task in setting up the nanopore transduction detector. The sensitivity of an optimally designed wrench to changes in it's environment can be several magnitudes greater than most molecular "nanopore-epitope" captures, as such it introduces a critical gain to the device, enabling a remarkable capability. The first step in the new detection process is that the auxiliary molecule is linked to a molecule external to the channel, and the externally-linked molecule and its binding interactions remain a significant part of the auxiliary molecules sensing "environment" (according to the design optimization). With the heightened sensitivity, the nanopore transduction apparatus provides a coupling mechanism that can transduce *single-molecule *events/states into stationary statistics phase changes in the toggle signal of a linked auxiliary molecule.

The second step in the new process is the means to tap into the information-rich toggle of the auxiliary molecule that links into the system of interest. Changes in the statistical profiles of the blockade data (i.e., different phases of stationary statistics in the ionic current blockades) are ascertained with high sensitivity. The sensitivity is due to use of machine learning (artificial intelligence) methods to fully differentiate and adaptively track different blockade signals under device drift conditions, with strong methods for feature extraction, classification, and clustering. Part of this software also enables the device by allowing introduction of pattern recognition informed feedback sampling (as well as force-ramp analysis, etc.). The software design, to allow for *real-time *pattern recognition informed feedback, was recently applied to discriminate between two DNA molecules as they were observed (typically within a few hundred milliseconds of onset of blockade, [1]). This capability alone can boost a nanopore detector's *productivity *by magnitudes, i.e., what if the signal of interest was one-in-a-thousand, if there were an active means to reject signals not of interest, and allow most of the signal acquisition time to focus on the signals of interest, this could increase acquisition approx. a thousand-fold on signal of interest.

**Figure 4:**
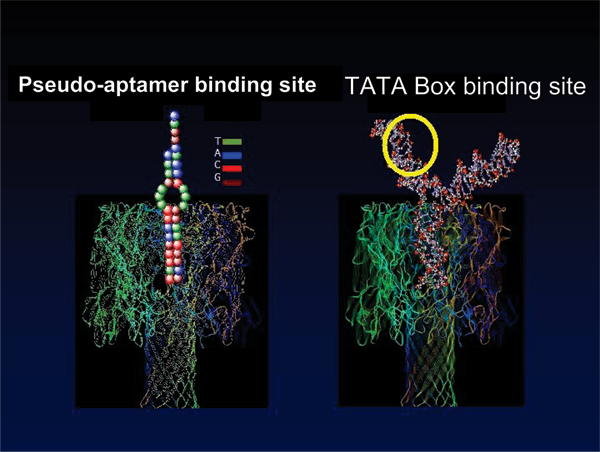
**DNA/RNA bifunctional transduction molecules**. The molecules shown were designed to help in the examination of DNA-DNA binding (left panel, with introduction of 5'-TACCT-3' annealing complement, see [5] for details) and DNA-protein binding (right panel, DNA Y-aptamer with TATA binding site, examined upon introduction of TATA Binding Protein, see [4] for details). Each molecule is designed to have a length of blunt-ended duplex DNA that is to be captured by the channel, that length is "terminated" such that the captured end is perched directly above the limiting aperture of the channel, free to move, bind to channel, and un-bind, in the high electrophoretic field strength concentration at the limiting aperture. For the termination with 3 T mismatch "bulges", a length of 9 base-pairs suffices for good channel blockade modulation; for termination at the Y-branching chosen, a length of 10 base-pairs works best.

**Figure 5:**
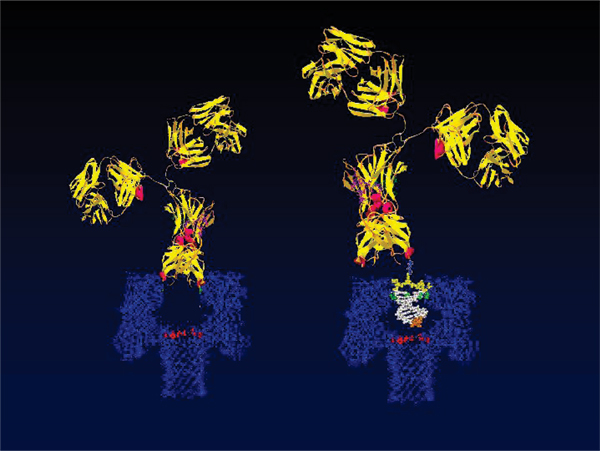
**Antibody-based bifunctional transduction molecules**. Antibody can generally function alone as bifunctional transducer. Drawback – *multiple *nanopore-epitopes (i.e., capture orientations, see Fig. 6), strength – possibly better signal change w/wo binding on a particular capture orientation (see Fig. 7)

In the experiments described in [3-5] the molecules with binding of interest (antibodies and aptamers) themselves produce the very sensitive, rapidly changing, blockade signal due to their interaction kinetics with the channel environment – thus bifunctional in binding and desirable interaction kinetics (see Fig. 4 and the left-side on Fig 5). The nanopore transduction results described here, however, are obtained from measurements of channel current blockades from a specially selected, partly channel-captured, ***auxiliary molecule***, that is rigidly/covalently bound (linked) to the molecule of interest (see Fig. 5, right-side), and then exposed to a solution containing the other molecule of interest. The transitions between different stationary phases of blockade can then be related to the bound/unbound configuration between the two molecules of interest to reveal their binding kinetics (and binding strength). This is a much more generalizable platform and demonstrates the general applicability of this mechanism for observation of single-molecule events.

**Figure 6:**
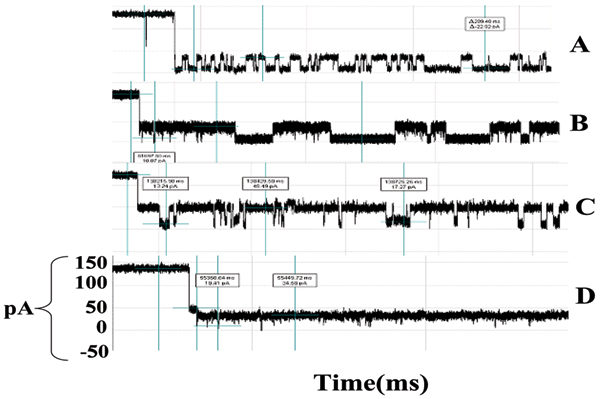
**Multiple antibody blockade signal classes**. A-D:Examples of the various IgG captures and their associated toggle signals. All share the same pA axis, all traces are for 1 second.

**Figure 7:**
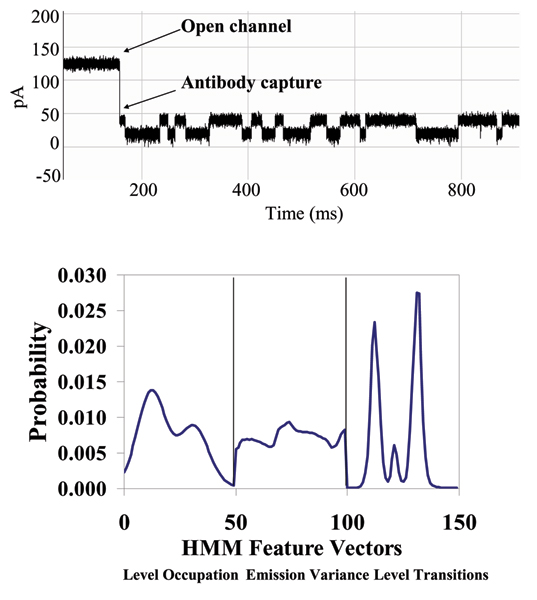
**Example that provides a very clear, stable, blockade direct by an Ab**. **Top: **A toggle signal is generated as a channel-captured region of the molecule (IgG) wiggles above the limiting aperture of the alpha-hemolysin channel varying the ionic current between two transient states. **Bottom: **Antibody Toggle HMM Signal Profile. The 150 feature vectors obtained from the 50-state HMM-EM/Viterbi implementation in [6-11] are: the 50 dwell percentage in the different blockade levels (from the Viterbi trace-back states), the 50 variances of the emission probability distributions associated with the different states, and the 50 merged transition probabilities from the primary and secondary blockade occupation levels (fits to two-state dominant modulatory blockade signals).

The blockade signals for a murine IgG1 monoclonal antibody, directed against the synthetic polypeptide antigen (Y,E)-A--K have been examined in prior work [41]. The antibody preparation was grown in hybridoma supernatant and purified by antigen affinity chromatography, and thus represents a highly purified population of antibody. Upon introduction into the chamber, the antibody exhibits more than one blockade signal (see Fig. 6), suggesting that the different parts of the molecule can be drawn into the channel. Fig. 7 shows a very stable channel blockade signal resulting from this setup, this and further descriptions of the IgG experiments are provided in [3] (this Journal). (The antibody-antigen system examined for the linked antibody described in the Results consists of an inexpensive antibody that binds to biotin.) Since the immunoglobulin domain fold is an elongated barrel, it is possible that capture events could occur at the narrow ends of the domain, as individual chains, or portions of both chains, within the Fab and Fc, and most likely, the carboxy terminus of the heavy chain (see Fig. 5). Similar findings, have been observed with other purified antibodies.

Molecular recognition and Antibody-Antigen interaction, in particular, depends strongly on buffer and temperature conditions [42]. The interaction can be affected by small ions, which can compete for the charge centers on the active cite surface, attenuating the interaction. In presence of chaotropic agents a protein molecule makes conformational changes; similar changes may be caused by a sufficient pH shift or by denaturing agents. A nanopore detector provides new opportunities to study protein-protein(ligand) reaction (antibody-antigen reaction, in particular) [3]. The nanopore formed by alpha-Hemolysin is quite sensitive to environmental conditions, as well, so care must be taken to examine the influence of buffer composition on the nanopore channel properties themselves. Preliminary work along these lines is given in the Results and further work along these lines can be found in [3].

The transduction detection method, thus, holds great promise for detailed study of (i) individual multi-component molecular complexes, (ii) conformational change and folding during the forming of such complexes, and (iii) enzyme study in general. All cases have immense potential in the area of drug design, particulary the role of designing better co-factors and adjuvants.

### Machine-Learning based cheminformatics

The entry of computational methods into biology, medical science, and chemical engineering has grown more significant in recent years with advances in practical, deployable, methods for pattern recognition and knowledge discovery. With use of more sophisticated computational modeling there is no need to restrict to fully parameterized, easily managed, mathematical expressions. A computer can now track all data instances directly with no need to fit to a parametric model. Support Vector Machines (SVMs), for example, are often used for classification due to their highly accurate performance. A growing trend of many multidisciplinary computational endeavors is that highly accurate, adaptive, and possibly unsupervised informatics approaches are *critically required *to boost experimental control and sensitivity, such that the research endeavor or technological method can become workable in practice. Such informatically leveraged research efforts are at a multidisciplinary nexus of scientific and technological applications.

Hidden Markov Models (HMMs) [40] provide a statistical framework for sequences of observations obeying Markov statistics. HMMs are excellent for identifying structure in sequential information, such as in channel current blockades or gene structure identification. Novel hash-interpolating and gap-interpolating Markov models are introduced in [43]. The standard HMM implementation inherits a length distribution on its same-state regions according to the memoryless probability that it be in those regions (from stationarity). Thus, a geometric distribution results on length distributions of same-state regions in the standard HMM (such as for exon or intron regions in gene finding). The geometric distribution is often very inadequate to describing the true distribution on the data, however, particularly for the very short and very long duration events where the true distribution typically deviates from the geometric distribution the most. An HMM that also models true length distribution information is known as an HMM-with-Duration. Unfortunately, the algorithms available for an HMM-with-Duration are very difficult to implement and are computationally expensive. In recent work [1,43,44], however, a new form of HMM-with-Duration is described with an algorithmic solution at the level of the column processing in the dynamic programming table constructed during the Viterbi calculation. This has far-reaching application and will be a powerful tool for research in Nanopore Detector Cheminformatics and gene structure identification. For channel current data a key objective is to get kinetic information. In nanopore blockade detection this information can be directly obtained from the dwell-times in the different blockade levels. This is generally a critical feature extraction regardless. To this end a method has been developed to filter the blockade pattern such that the major transitions between blockade levels are *strongly preserved *(EVA projection, see [43]), but such that the minor (noise) transitions within a blockade level project to the means of their dominant level. The problem when this approach is pushed to an extreme, or the noise level is increased sufficiently, is that spurious transitions between the major levels begin to occur – which corrupts the kinetic analysis. The solution is to use an HMM with Duration formalism in conjunction with the EVA projection method (see [1], for more Background and Results).

**Figure 8:**
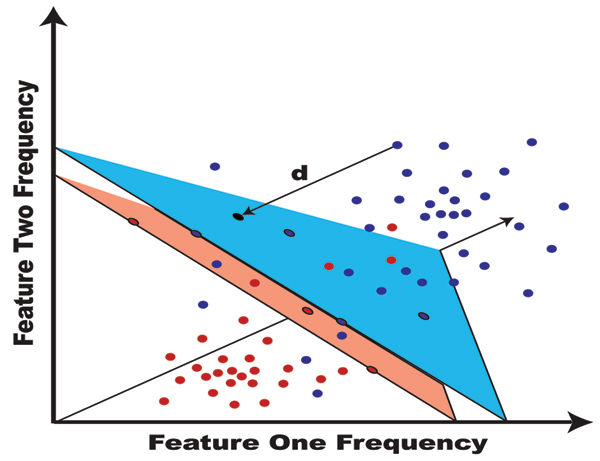
SVM: hyperplane separability, with a margin.

Support Vector Machines (SVMs) are variational-calculus based methods that are constrained to have structural risk minimization (SRM), unlike neural net classifiers, such that they provide noise tolerant solutions for pattern recognition [45,46]. Simply put, an SVM determines a hyperplane that optimally separates one class from another (see Fig. 8). Once learned, the hyperplane allows data to be classified according to the region (separated by the hyperplane) in which it resides. Currently there are two approaches to implementing *multiclass *SVMs. One arranges several *binary *classifiers as a decision tree such that they perform a multi-class decision-making function (SVM-external classification). The second approach involves solving a single optimization problem corresponding to the entire data set (with multiple hyperplanes), with multi-class discriminator optimization performed internally. The SVM-internal approach, when it is stable and properly generalizable (an area of ongoing research), is preferred, since a tuning over Decision tree topologies and weightings is avoided [47].

Each SVM approach encapsulates a significant amount of model-fitting information in its choice of kernel. In some sense, the SVM kernel provides a notion of distance to the decision hyperplane. In prior work, novel, information-theoretic, kernels were successfully employed for notably better performance over standard kernels [8,47]. In situations where the data isn't clearly separable, making for poor discrimination, signal clustering is used to provide robust and useful information – to this end, novel, SVM-based clustering methods have been introduced as well [47,48] (as with classification, Internal and External SVM Clustering algorithms have been explored).

Thus, SVMs are fast, easily trained, discriminators [45,46], for which strong discrimination is possible without the over-fitting complications common to neural net discriminators [45]. In application to channel current signal analysis there is generally an abundance of experimental data available, if not, the experimenter can usually just take more samples and make it so. In this situation it is appropriate to seek a method good at both classifying data and evaluating a confidence in the classifications given. In this way, data that is low confidence can simply be dropped. The structural risk minimization at the heart of the SVM method's robustness also provides a strong confidence measure. For this reason, SVM's are the classification method of choice for channel current analysis, as they have excellent performance at 0% data drop, and as weak data is allowed to be dropped, the SVM-based approaches far exceed the performance of other methods.

In [8], novel, information-theoretic, kernels were first introduced for notably better performance over standard kernels – where discrete probability distributions comprised part of the feature vector data. The use of probability vectors, and L_1_-norm feature vectors in general, turns out to be a very general formulation, wherein feature extraction makes use of signal decomposition into a complete set of separable states that can be interpreted or represented as a probability vector (or normalized collection of such, etc.). A probability vector formulation also provides a straightforward hand-off to the SVM classifiers since all feature vectors have the same length with such an approach. What this means for the SVM, however, is that geometric notions of distance are no longer the best measure for comparing feature vectors. For probability vectors (i.e., discrete distributions), the best measures of similarity are the various information-theoretic divergences: Kullback-Leibler, Renyi, etc. By symmetrizing over the arguments of those divergences a rich source of kernels is obtained that works well with the types of probabilistic data obtained, as shown in [8,47].

### Summary of preliminary binding studies results

**Figure 9:**
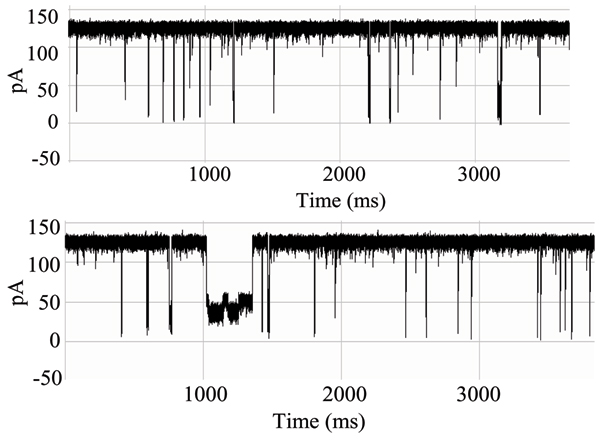
**Pseudo-aptamer study with DNA overhang binding complement – background blockades**. Top: Un-annealed ssDNA translocation blockades. Bottom: Possible instance of reverse-oriented capture/melting.

**Figure 10:**
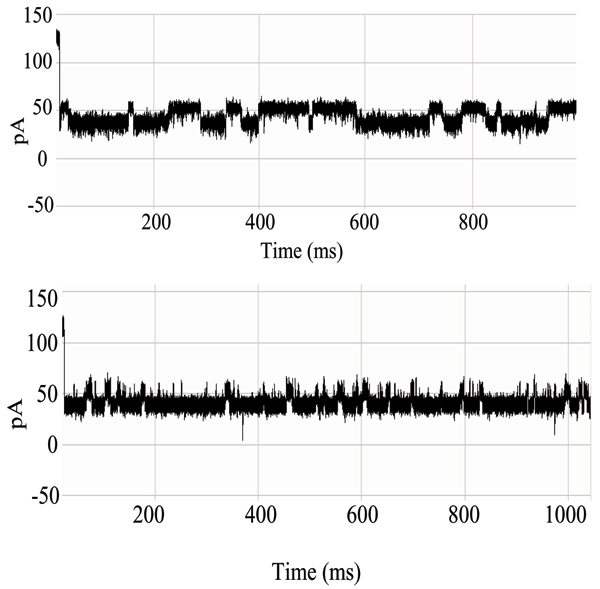
**Pseudo-aptamer: DNA overhang binding complement – signal blockades**. **Top: **Before introduction of 5-base ssDNA complement.**Bottom: **After introduction of complement.

Binding analysis with aptamers is much easier than with antibody-antigen due to the strong charge distributed along a DNA molecule, leading to a much stronger electrophoretic force interaction. The problem is that this force can be too strong when the molecule is actually captured, stifling sensitive blockade transitions. In all the engineered molecules described here, however, the pseudo-aptamers have bulges, loops, or Y-branching geometries to perch the terminal base-pair similar to that of the DNA control hairpins studied in [8,10,11]. Figures 9 and 10 show results for pseudo-aptamers involving DNA molecules with single-stranded overhangs (designed to examine binding with the complement stands to the overhangs). The goal is to eventually extend to the ssDNA overhangs to ssDNA links to a SELEX identified ssDNA aptamer region (for ssDNA/RNA-type aptamers).

**Figure 11:**
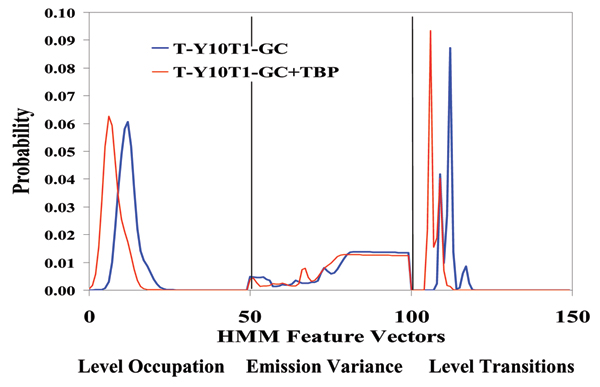
**Y-aptamer, with TATA Box, that binds TBP**. Red curve: Profile of Y-aptamer signal blockades before introduction of TBP. Blue curve: Y-aptamer signal blockade after introduction of TBP. A drastically different signal profile, possibly indicative of significant conformational change in the Y-aptamer upon TBP binding.

**Figure 12:**
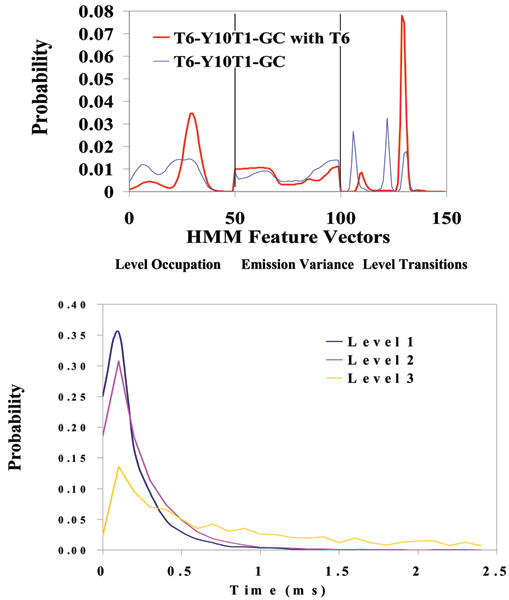
**Y-aptamer with DNA overhang that binds complement**. Top: signal profiles before and after binding. Bottom: the dwell-time distributions on the three dominant levels indicated in the *unbound *blockade signal. The profiles are surprisingly different, the bound case, with annealed complement, appears to be more "stable", with only two dominant blockade levels. This is consistent with it being a molecule with fewer degrees of freedom (with 6 T overhang now annealed to 6 A complement).

Synthetic transcription factors (STFs) promise to offer a powerful new therapeutic against Cancer, AIDS, and genetic disease. STFs that can appropriately target (and release) their transcription factor binding sites (TFBS) on native genomic DNA provide a means to directly influence cellular mRNA production (to induce death or dormancy for Cancer and AIDs cells, or restore proper cellular function in the case of genetic disease). In synthetic TF drug discovery an effective mechanism for screening amongst TF candidates would itself be highly valued. Such may be possible with novel observation and analysis methods involving channel current observations of single molecule interactions/blockades. Figures 11 and 12 describe results having to do with such efforts, with a Y-aptamer encoded with the transcription factor binding site of interest (a TATA box) and the binding partner TATA binding protein (TBP).

#### Antibody-Antigen Binding/Biosensing

**Figure 13:**
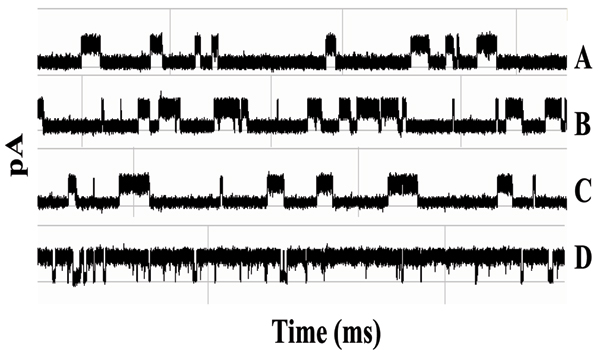
**Antibody-Antigen binding – clear example from specific capture orientation**. A region of antibody molecule has inserted into the Alpha-hemolysin channel to produce a constant toggle signal. Antigen is introduced in frame (A). Sub-sequential data files containing toggle signals of three minute intervals are recorded and displayed as B, C, and D. Changes to the toggle signal are detected in frames C and D indicating the binding event between the antibody and antigen has taken place.

It is found that the antibody blockade signal alters shortly after introduction of antigen, as Fig. 13 shows upon addition of a moderately high concentration (100 μg/ml) of 200 kD multivalent synthetic polypeptide (Y,E)-A--K. Presumably, these changes are the result of antibody binding to antigen. The time before the blockade signal is altered is also interesting; it ranges from seconds to minutes (not shown). This presumably is a reflection of antibody affinity.

## Results

### DNA/biotin-streptavidin binding experiment

**Figure 14:**
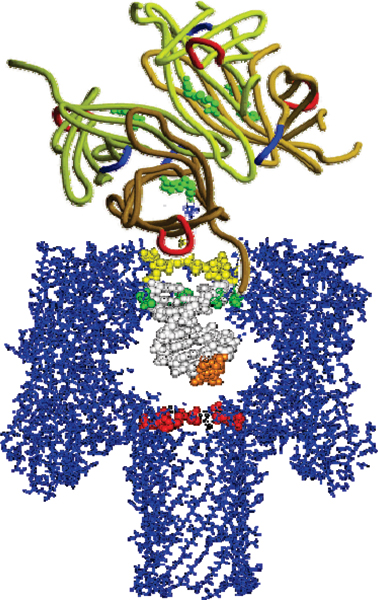
**Biotinylated DNA hairpin binding streptavidin**. A DNA hairpin "gauge" is designed to offer a clear multi-level modulated blockade signal. The DNA gauge has it's hairpin loop section altered from the 4 dT loop used in the controls, the loop has a linkage to biotin placed in the middle of it's 4 dT loop (see Methods). One of these DNA hairpins with attached biotin is captured in the channel, an excess of Streptavidin is introduced. Transition to a new blockade state is quickly seen, one only seen after introduction of streptavidin.

**Figure 15:**
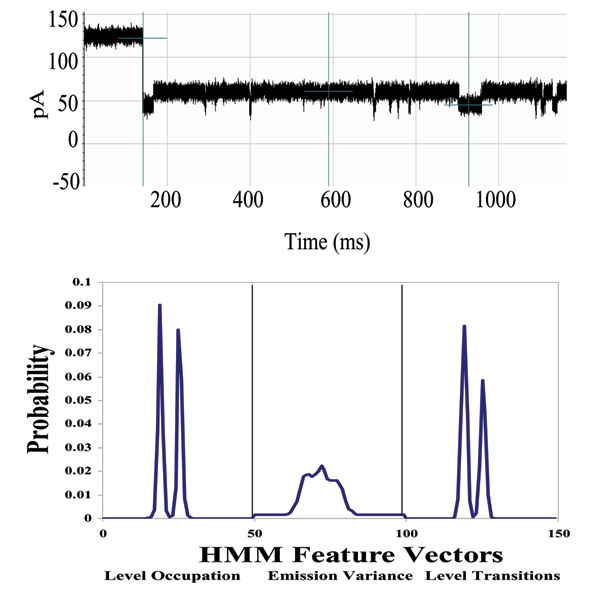
**Biotinylated DNA hairpin blockade signal and profile**. (See Fig. 7 or Methods for description of HMM profile.)

**Figure 16:**
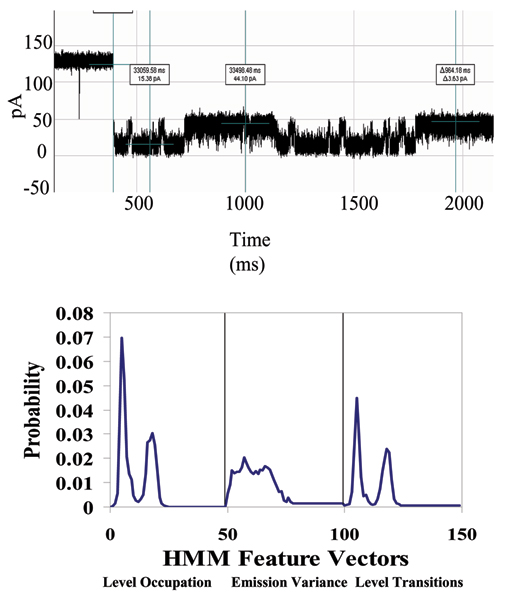
**Biotinylated DNA hairpin capture after introduction of an excess of streptavidin – significantly altered blockade signal and profile**. (See Fig. 7 or Methods for description of HMM profile.)

A series of nanopore experiments is performed with blockade signal transduction on the DNA/biotin-streptavidin system described in Fig. 14. In those experiments a hairpin is used that is a variant of the familiar 9 base-pair DNA hairpins that are used as controls, via attachment of a biotin linker via a modified thymine at the top of the hairpin loop (3'-side). The modification introduces a six carbon spacer arm that extends perpendicularly to the axis of the DNA. This allows for sterically unhindered biotin interaction. The binding partner introduced later is streptavidin to have as control the strongest interaction known in biology (biotin-streptavidin). Clear indications of new blockade signal states, only after introduction of streptavidin, has been obtained. Signal analysis focusing on these blockades of interest is shown in Figures 15 and 16.

### DNA/antibody-biotin binding experiment

**Figure 17:**
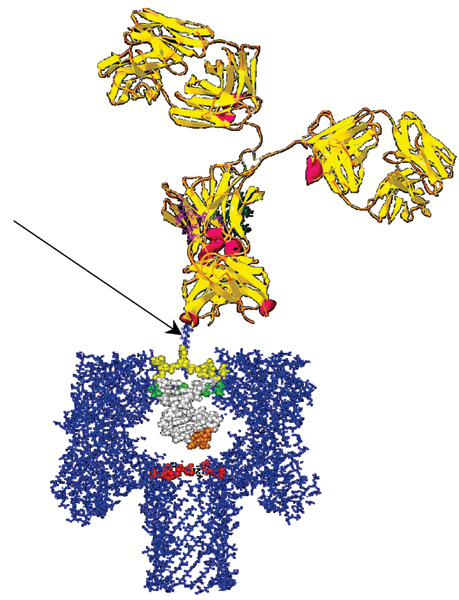
**DNA hairpin bound to antibody via an EDC-linker**. Approximately shown to scale. Arrow points to the Internal Amino Thymine Modification with Primary Amine on a six carbon spacer arm. Primary amine can be crosslinked using 1-Ethyl-3-(3-dimethylaminopropyl) carbodiimide hydrochloride (EDC) to the peptide carboxyl terminus of the antibody heavy chain. This crosslinkage results in a covalent bond between the primary amine and the carboxyl.

**Figure 18:**
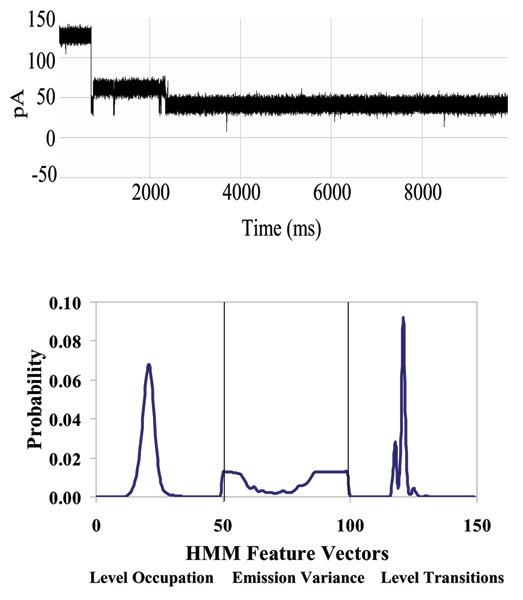
**Antibody linked to DNA-hairpin blockade signal and HMM profile**. (See Fig. 7 or Methods for description of HMM profile.)

**Figure 19:**
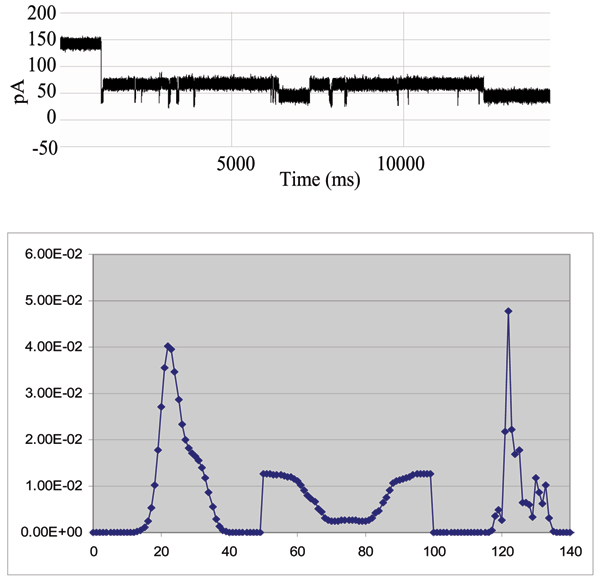
**Antibody linked to DNA-hairpin, now bound to its target antigen (biotin) – new blockade signal, and associated HMM profile**. Antigen binding to an EDC-linked Antibody/DNA-Hairpin, where stem of the hairpin is captured in the Nanopore Detector. (See Fig. 7 or Methods for description of HMM profile.)

The general-case nanopore transduction system, that involving linking a molecule with binding properties of interest is studied next. The system consists of biotin-binding antibody that is linked to a specially designed DNA-based channel current modulator (see Fig. 17). It has been hypothesized that binding to the molecule of interest might then result in a change in the channel modulator's stationary statistics, and this is shown in Figures 18 and 19. Antigen alone is introduced to the channels, as well as non-specific binding contaminants, none give rise to the new classes of lengthy dwell-time blockade events that are seen. These and other controls strongly indicate that the on-binding interactions are being seen between the antibody and its biotin target. What is not being seen are the off-binding events of the biotin dissociating from the antibody. This is consistent with the strength of the binding and the short lifetime (hours) of the experimental window. What is pursued on bringing these time-scales into agreement is introduction of MgCl_2 _to weaken the antibody-biotin binding strength (where weakening the DNA-channel interactions isn't so critical, as long as the modulator exhibits difference between the bound and unbound cases).

### α-Hemolysin channel stability with different buffer conditions

The channel proved to be stable to high salt concentrations (MgCl_2 _above 2 M and KCl up to 4 M) and presence of some other additives (urea 2 M, glycerol 5%) at pH around 8.0. Typical pattern of current rise with increase in background electrolyte, KCl is shown in the figure in Additional File 1. Specifically, the current versus KCL concentration is shown in running buffer with composition 1 M KCl, 20 mM HEPES (pH 8.0), with HEPES concentration maintained constant as content of KCl is increased. The total resistance vs. MgCl_2 _is shown in the figure in Additional File 2. Titration with 4 M MgCl_2 _is shown, and **c**urrent through the α-HL channel increases as electrolyte concentration rises. Initial concentration of background electrolyte, MgCl_2_, is 1 Mol/L.

The curves in those figures demonstrate the appropriate current increase with further tendency of its stabilization at high salt concentration as it is expected for conductivity behavior of electrolyte solution. We observed considerable asymmetry in conductance for the cases when the positive and negative potential applied, and results for this are shown in the figure in Additional File 3, which provides the total resistance vs. MgCl_2_, with MgCl_2 _concentration increase. The titration is performed with 2 M MgCl_2 _dissolved in background electrolyte, 1 M KCl. The upper and lower curves correspond to the negative and positive applied potentials, accordingly.

One possible explanation is presence of the charged amino acid residues on the inner surface of the protein channel that may contribute such an asymmetry by different mechanisms, although we have to emphasize that experimental data are to be related to the integral resistance of the system rather than a single channel. Therefore, the value for channel resistance, obtained by the ratio of the applied potential to the measured current, may be overestimated. The conductance asymmetry decrease with increasing ionic strength could relate to surface charge shielding, or to possible electroosmotic transport effects.

### DNA-hairpin blockade signal stability with different buffer conditions

An increase in concentration of background electrolyte, KCL, generally provides only moderate influence on the current blockade signal. This is shown in the figure in Additional File 4, where current blockade signal change is examined with KCl increase. In that figure a nine base-pair DNA hairpin (ULT-HP) with a distinctive upper level toggle is shown in the top panel. The middle and bottom panels show the blockade patterns at 1.9 and 2.5 M KCl, correspondingly. As the concentration of background electrolyte increases, the signal keeps its highly structured profile, although becoming noisy at KCL concentrations above 2 M (panels 2 and 3 in the figure). In the figure in Additional File 5, the 150 feature profile [2] of the data is shown at 1.0, 1.9 and 2.5 M KCl (corresponding to the signal shown in the previous image). Each blockade signature is de-noised by 5 rounds of Expectation-Maximization (EM) training on the parameters of the HMM. After the EM iterations, 150 parameters are extracted from the HMM. The 150 feature vectors obtained from the 50-state HMM-EM/Viterbi implementation in are: the 50 dwell percentage in the different blockade levels (from the Viterbi trace-back states), the 50 variances of the emission probability distributions associated with the different states, and the 50 merged transition probabilities from the primary and secondary blockade occupation levels (fits to two-state dominant modulatory blockade signals). Other preliminary work with introduction of chaotropic agents is described in [3].

## Discussion/conclusion

### Nanopore detection

What is shown here is initial verification of a general form of a new event transduction mechanism. Single-molecule biochemical analysis using channel current transduction cheminformatics appears to be a viable technology. To maintain the existing biologically-based channel, however, there are salt and pH restrictions on the analyte solutions. More versatility is clearly needed here, but the significant strengths of this approach, with informatics methods leveraging the useful sensitivity of the detector, suggest great potential utility in this approach.

The examination of transcription factor binding to target transcription factor binding site (TF/TFBS interactions) affords the possibility to understand, quantitatively, much of the Transcriptome. This same information, coupled with new interaction information upon introduction of synthetic TFs (possible medicines), provides a very powerful, directed, approach to drug discovery.

Upon binding to antigen, a series of events are initiated by the interaction of the antibody carboxy-terminal region with serum proteins and cellular receptors. Biological effects resulting from the carboxy-terminal interactions include activation of the complement cascade, binding of immune complexes by carboxy-terminal receptors on various cells, and the induction of inflammation. Nanopore Detection provides a new way to study the binding/conformational histories of individual antibodies. Many critical questions regarding antibody function are still unresolved, questions that can be approached in a new way with the nanopore detector. The different antibody binding strengths to target antigen, for example, can be ranked according to the observed lifetimes of their bound states. Questions of great interest include: are allosteric changes transmitted through the molecule upon antigen binding? Can effector function activation be observed and used to accelerate drug discovery efforts?

Thus, real-time analysis of antibody IgG binding affinity might be possible using a nanopore detector to better understand antibody-antigen binding affinities and the conformational changes that initiate signal pathways.

In the figure in Additional File 6 is shown the automatically generated plot of spike characteristics for blockade data when DNA hairpins were examined, one radiated and one not. Spike count plots are generated to show increasing counts as cut-off thresholds are relaxed (to where eventually any downward deflection will be counted as a spike). The plots are automatically generated and automatically fit with extrapolations of their linear phases (exponential phases occur when cut-offs begin to probe the noise band of a blockade state – typically gaussian noise "tails"). The extrapolations provide a stable, "robust", estimate of anomalous spike counts. By this method, the non-radiated DNA exhibited a full-blockade "spike" from its lower-level blockade with a frequency of 5 spikes per second (indicating a fraying of the blunt ended terminus of the molecule at that rate). For the radiated molecule the frequency of spikes was 15 spikes per second, indicating a much greater fraying rate (dissociation of the terminal base-pair), consistent with that molecule being weakened by radiation such that it's terminal base-pair frays more frequently.

### Channel current cheminformatics

Web-accessible tools for HMM-based feature extraction and SVM classification are accessible at  (see [1], for latest web-interface discussion). The web tools can help in identification of blockade levels, the level transition and lifetime characteristics, and the fast blockade "spike" characteristics. The SVM classification is of general use for any kind of classification problem, and a number of novel kernels and novel implementation are employed. SVM-based clustering is also implemented in a novel way to yield a non-parametric clustering approach, which is used to cluster signals into multiple classes (particularly important for complex multi-orientation data-sampling situations such as with an antibody). The Web interface also provides access to several SVM variants that show significantly improved performance over the standard Platt-SMO implementation with a Gaussian kernel. The methods involve novel kernels designed to measure "distance" not in a geometric sense but in a probability measure sense.

An expert-mode interface with up-loadable Perl modules for kernels and alpha-selection heuristics is in development. The interface to this and all other software described is available via the webpage: . As with the multiclass SVM discriminator implementations, the strong performance of the binary SVM enables SVM-External as well as SVM-Internal approaches to clustering. The external-SVM clustering algorithm introduced in [47] clusters data vectors with no *a priori *knowledge of each vector's class. The algorithm works by first running a Binary SVM against a data set, with each vector in the set randomly labeled, until the SVM converges. In order to obtain convergence, an acceptable number of KKT violators must be found. This is done through running the SVM on the randomly labeled data with different numbers of allowed violators until the number of violators allowed is near the lower bound of violators needed for the SVM to converge on the particular data set. Choice of an appropriate kernel and an acceptable sigma value also will affect convergence. After the initial convergence is achieved, the (sensitivity+specificity) will be low, likely near 1. The algorithm now improves this result by iteratively re-labeling the worst misclassified vectors, which have confidence factor values beyond some threshold, followed by rerunning the SVM on the newly relabeled data set. This continues until no more progress can be made. Progress is determined by an increasing value of (sensitivity+specificity), hopefully nearly reaching 2. This method provides a way to cluster data sets without prior knowledge of the data's clustering characteristics, or the number of clusters.

An exciting area of machine learning research is being brought to bear on the kinetic signal decomposition of channel currents. The external-SVM approach described in the Background and [47] offers to provide one of the most powerful, unsupervised, methods for clustering. Part of the strength of the method is that it is non-parametric. Part of the weakness is in obtaining an initial clustering. To improve on this, attempts have been made to graft the external-SVM onto an initial clustering using bisect-K-means (that is seeded by principle direction divisive partitioning, or principle component analysis, when random seeding does poorly). Other grafts have been tried as well, what is commonly seen is that such grafting inherits the local minima traps of the grafting parent. For this reason, the delay on initial convergence of the non-parametric method may be a mixed blessing in that it truly stands to offer something new. External-SVM clustering, along the lines of [47], may allow precise cluster re-growth by its ability to operate on a shifting, optimally-defined boundary region (via retraining of support vector structure) using direct label operations (binary "flipping" – see recent Results and Discussion in [48]).

## Methods

### Nanopore experiments

Each experiment is conducted using one α-hemolysin channel inserted into a diphytanoyl-phosphatidylcholine/hexadecane bilayer across a 25-micron-diameter horizontal Teflon aperture, as described previously [8,11] (Fig. 1). Seventy microliter chambers on either side of the bilayer contains 1.0 M KCl buffered at pH 8.0 (10 mM HEPES/KOH) except in the case of buffer experiments where the salt concentration, pH, or identity may be varied. Voltage is applied across the bilayer between Ag-AgCl electrodes. DNA control probes are added to the *cis *chamber at 10 or 20 μM final concentration. All experiments are maintained at room temperature (23 ± 0.1°C), using a Peltier device.

### Control probe design

Since the five DNA hairpins studied in the prototype experiment have been carefully characterized [8], they are used in the antibody (and other) experiments as highly sensitive controls. Use of the controls entails testing a channel, especially an oddly behaving channel, with a known nine base-pair DNA hairpin control. If the familiar, visibly discernible, control blockade signals doesn't occur, the channels viability is then looked into further. The nine base-pair hairpin molecules examined in the prototype experiment share an eight base-pair hairpin core sequence, with addition of one of the four permutations of Watson-Crick base-pairs that may exist at the blunt end terminus, i.e., 5'-G•C-3', 5'-C•G-3', 5'-T•A-3', and 5'-A•T-3'. Denoted 9GC, 9CG, 9TA, and 9AT, respectively. The full sequence for the 9CG hairpin is 5' CTTCGAACGTTTTCGTTCGAAG 3', where the base-pairing region is underlined. The eight base-pair DNA hairpin is identical to the core nine base-pair subsequence, except the terminal base-pair is 5'-G•C-3'. The prediction that each hairpin would adopt one base-paired structure was tested and confirmed using the DNA mfold server . A standardized aliquot of antibody is used as the control for antibody experiments once the kinetics of antibody capture and antigen-binding events are established and shown to be highly reproducible.

### Anti-biotin antibody, used w/wo linkage to a DNA hairpin nanopore-probe

Experimental setup is described in detail in [3]. Anti-biotin monoclonal antibodies obtained from Stressgen (San Diego, California) were used for binding studies. The antibodies, stored at -20 C as supplied, were brought to a final dilution 1–4 μg/mL in the electrolyte chamber. Ab-DNA conjugation was performed with 1-Ethyl-3-(3-Dimethylaminopropyl) carbodiimine Hydrochloride (EDC), in accordance with the instructions of Manufacturer (Pierce, Rockford, IL). Potassium chloride, HEPES and magnesium chloride were purchased from Sigma, St. Louis, MO. Other chemicals were from Fisher Scientific, Atlanta, GA.

### Data acquisition

Data is acquired and processed in two ways depending on the experimental objectives: (i) using commercial software from Axon Instruments (Redwood City, CA) to acquire data, where current was typically be filtered at 50 kHz bandwidth using an analog low pass Bessel filter and recorded at 20 μs intervals using an Axopatch 200B amplifier (Axon Instruments, Foster City, CA) coupled to an Axon Digidata 1200 digitizer. Applied potential was 120 mV (*trans *side positive) unless otherwise noted. In some experiments, semi-automated analysis of transition level blockades, current, and duration were performed using Clampex (Axon Instruments, Foster City, CA). (ii) using LabView-based experimental automation. In this case, ionic current was also acquired using an Axopatch 200B patch clamp amplifier (Axon Instruments, Foster City, CA), but it was then recorded using a NI-MIO-16E-4 National Instruments data acquisition card (National Instruments, Austin TX). In the LabView format, data was low-pass filtered by the amplifier unit at 50 kHz, and recorded at 20 μs intervals. In both fixed duty cycle (i.e., not feedback controlled) data acquisition approaches, the solution sampling protocol used periodic reversal of the applied potential to accomplish the capture and ejection of single biomolecules. The biomolecules captured consisted of antibodies and antigen, bound together or not, and in various orientations, and DNA control probes with stem-capture orientation and were added to the cis chamber typically in 20 μM concentrations.

### Nanopore detector augmentation using bifunctional molecules

Nanopore Detector augmentation with bifunctional molecules brings a novel modification to the nanopore detection methodology. Now the idea is that the bifunctional auxiliary molecules produce a "toggling" blockade between several different levels (with two usually dominating). In other words, the resulting blockade signal for the auxiliary molecule by itself is no longer at approximately a fixed blockade level, but now consists of a telegraph-like blockade signal with stationary statistics. Upon binding of analyte to the auxiliary molecule (a binding site, or moiety, being its other functionality) the toggling channel blockade signal is greatly altered, to one with different transition timing and different blockade residence levels. Building on this as a binding affinity testing and biosensing platform requires sophisticated computational tools, such as Hidden Markov Models and Support Vector Machines, but offers at least a hundred-fold improvement to the sensitivity of the device. Given the noise in the system and the limited dynamic range for blockades of the open channel current, the device is greatly restricted if not endowed with the sensitive timing information. It has even been found that minor environmental alterations to temperature, pH, etc., results in the toggle signal produced by "toggling" type auxiliary molecule being modified significantly – in essence the channel with toggling-type auxiliary molecules can provide sensitive biosensing on the solution environment itself.

### Channel current signal analysis & pattern recognition

A Channel Current Spike Detector algorithm was developed in [8] to characterize the brief, very strong, blockade "spike" behavior observed for molecules that occasionally break in the region exposed to the limiting aperture's strong electrophoretic force region. (In [6-11], where nine base-pair hairpins were studied, the spike events were attributed to a fray/extension event on the terminal base-pair.) Together, the formulation of HMM-EM, FSAs and Spike Detector provide a robust method for analysis of channel current data. The spike detector software is designed to count "anomalous" spikes, i.e., spike noise not attributable to the gaussian fluctuations about the mean of the dominant blockade-level. Spike count plots are generated to show increasing counts as cut-off thresholds are relaxed (to where eventually any downward deflection will be counted as a spike). The plots are automatically generated and automatically fit with extrapolations of their linear phases (exponential phases occur when cut-offs begin to probe the noise band of a blockade state – typically gaussian noise "tails"). The extrapolations provide an estimate of "true" anomalous spike counts (see figure in Additional file 6).

The signal processing architecture (Fig. 2) is designed to rapidly extract useful information from noisy blockade signals using feature extraction protocols, wavelet analysis, Hidden Markov Models (HMMs) and Support Vector Machines (SVMs). For blockade signal acquisition and simple, time-domain, feature-extraction, a Finite State Automaton (FSA) approach is used [19] that is based on tuning a variety of threshold parameters. A generic HMM can be used to characterize current blockades by identifying a sequence of sub-blockades as a sequence of state emissions [6-9,11]. The parameters of the generic-HMM can then be estimated using a method called Expectation/Maximization, or 'EM" [40], to effect de-noising. The HMM method with EM, denoted HMM/EM, is used in what follows (further Background on these methods can be found in [6-11]). Classification of feature vectors obtained by the HMM for each individual blockade event is then done using SVMs, an approach which automatically provides a confidence measure on each classification.

The Nanopore Detector is operated such that a stream of 100 ms samplings are obtained. Each 100 ms signal acquired by the time-domain FSA consists of a sequence of 5000 sub-blockade levels (with the 20 μs analog-to-digital sampling). Signal preprocessing is then used for adaptive low-pass filtering. For the data sets examined, the preprocessing is expected to permit compression on the sample sequence from 5000 to 625 samples (later HMM processing then only required construction of a dynamic programming table with 625 columns). The signal preprocessing makes use of an off-line wavelet stationarity analysis (Off-line Wavelet Stationarity Analysis, Figure 2) to determine the amount of sample compression (effective low-pass filtering) that can be sustained and still have good structure resolution.

With completion of preprocessing, an HMM is used to remove noise from the acquired signals, and to extract features from them (Feature Extraction Stage, Fig. 2). The HMM is, initially, implemented with fifty states, corresponding to current blockades in 1% increments ranging from 20% residual current to 69% residual current. The HMM states, numbered 0 to 49, corresponded to the 50 different current blockade levels in the sequences that are processed. The state emission parameters of the HMM are initially set so that the state j, 0 <= j <= 49 corresponding to level L = j + 20, can emit all possible levels, with the probability distribution over emitted levels set to a discretized Gaussian with mean L and unit variance. All transitions between states are possible, and initially are equally likely. Each blockade signature is de-noised by 5 rounds of Expectation-Maximization (EM) training on the parameters of the HMM. After the EM iterations, 150 parameters are extracted from the HMM. The 150 feature vectors obtained from the 50-state HMM-EM/Viterbi implementation in [6-11] are: the 50 dwell percentage in the different blockade levels (from the Viterbi trace-back states), the 50 variances of the emission probability distributions associated with the different states, and the 50 merged transition probabilities from the primary and secondary blockade occupation levels (fits to two-state dominant modulatory blockade signals). Plots of the class-averaged 150-component feature vector profiles are shown in Fig.'s 7, 11, 12, 15, 16, 18, and 19.

The HMM-with-Duration implementation, described in [1,43,44], has been tested in terms of its performance at parsing synthetic blockade signals. The synthetic data used in [1,43,44] was designed to have two levels, with lifetime in each level determined by a governing distribution (Poisson and Gaussian distributions with a range of mean values were considered). The results clearly demonstrate the superior performance of the HMM-with-duration over its simpler, HMM without Duration, formulation. With use of the EVA-projection method this affords a robust means to obtain kinetic feature extraction. The HMM with duration is critical for accurate kinetic feature extraction, and the results in [1,43,44] suggest that this problem can be elegantly solved with a pairing of the HMM-with-Duration stabilization with EVA-projection.

In the implementation of the HMM-with-duration in [43], the transition probabilities for state 's' to remain in state 's', a "ss" transition can be computed as: Prob(ss | s_length _= L) = Prob(s_length _≥ L + 1)/Prob(s_length _≥ L). The transition probabilities out of state 's' can have some subtleties, as shown in the following where the states are exon (e), intron (i), and junk (j). In this case, the transition probabilities governing the following transitions, (jj) -> (je), (ee) -> (ej), (ee) -> (ei), (ii) -> (ie) are computed as: Prob(ei | e_length _= L) = Prob(e_length _= L)/Prob(e_length _≥ L) × 40/(40 + 60) and Prob(ej | e_length _= L) = Prob(e_length _= L)/Prob(e_length _≥ L) × 60/(40 + 60), where the total number of (ej) transitions is 60 and the total number of (ei) transitions is 40. Further details, and recent results are described in [1].

The conventional HMM method is based on a stationary set of emission and transition probabilities. Emission broadening, via amplification of the emission state variances, is a filtering heuristic that leads to level-projection that strongly preserves transition times between major levels (see [43] for further details). This approach does not require the user to define the number of levels (classes). This is a major advantage compared to existing tools that require the user to determine the levels (classes) and perform a state projection. This allows kinetic features to be extracted with a "simple" FSA (Finite State Automaton) that requires minimal tuning. One important application of the HMM-with-duration method used in [43] includes kinetic feature extraction from EVA projected channel current data (the HMM-with-Duration is shown to offer a critical stabilizing capability in an example in [43]). The EVA-projected/HMMwDur processing offers a hands-off (minimal tuning) method for extracting the mean dwell times for various blockade states (the core kinetic information).

Binary Support Vector Machines (SVMs) are based on a decision-hyperplane heuristic that incorporates structural risk management by attempting to impose a training-instance void, or "margin," around the decision hyperplane [45,46]. Feature vectors are denoted by x_ik_, where index i labels the M feature vectors (1 ≤ i ≤ M) and index k labels the N feature vector components (1 ≤ i ≤ N). For the binary SVM, labeling of training data is done using label variable y_i _= ±1 (with sign according to whether the training instance was from the positive or negative class). For hyperplane separability, elements of the training set must satisfy the following conditions: w_β_x_iβ _- b ≥ +1 for i such that y_i _= +1, and w_β_x_iβ _- b ≤ -1 for y_i _= -1, for some values of the coefficients w_1_,...,w_N_, and b (using the convention of implied sum on repeated Greek indices). This can be written more concisely as: y_i_(w_β_x_iβ _- b) - 1 ≥ 0. Data points that satisfy the equality in the above are known as "support vectors" (or "active constraints").

Once training is complete, discrimination is based solely on position relative to the discriminating hyperplane: w_β_x_iβ _- b = 0. The boundary hyperplanes on the two classes of data are separated by a distance 2/w, known as the "margin," where w^2 ^= w_β_w_β_. By increasing the margin between the separated data as much as possible the optimal separating hyperplane is obtained. In the usual SVM formulation, the goal to maximize w^-1 ^is restated as the goal to minimize w^2^. The Lagrangian variational formulation then selects an optimum defined at a saddle point of L(w, b; α) = (w_β_w_β_)/2 - α_γ_y_γ_(w_β_x_γβ _- b) - α_0_, where α_0 _= Σ_γ_α_γ_, α_γ _≥ 0 (1 ≤ γ ≤ M). The saddle point is obtained by minimizing with respect to {w_1_,...,w_N_, b} and maximizing with respect to {α_1_,...,α_M_}. If y_i_(w_β_x_iβ _- b) - 1 ≥ 0, then maximization on α_i _is achieved for α_i _= 0. If y_i_(w_β_x_iβ _- b) - 1 = 0, then there is no constraint on α_i_. If y_i_(w_β_x_iβ _- b) - 1 < 0, there is a constraint violation, and α_i _→ ∞. If absolute separability is possible the last case will eventually be eliminated for all α_i_, otherwise it's natural to limit the size of α_i _by some constant upper bound, i.e., max(α_i_) = C, for all i. This is equivalent to another set of inequality constraints with α_i _≤ C. Introducing sets of Lagrange multipliers, ξ_γ _and μ_γ _(1 ≤ γ ≤ M), to achieve this, the Lagrangian becomes:

L(w, b; α, ξ, μ) = (w_β_w_β_)/2 - α_γ_[y_γ _(w_β_x_γβ _- b) + ξ_γ _] + α_0 _+ ξ_0_C - μ_γ_ξ_γ_, where ξ_0 _= Σ_γ_ξ_γ_, α_0 _= Σ_γ_α_γ_, and α_γ _≥ 0 and ξ_γ _≥ 0 (1 ≤ γ ≤ M).

At the variational minimum on the {w_1_,...,w_N_, b} variables, w_β _= α_γ_y_γ_x_γβ_, and the Lagrangian simplifies to: L(α) = α_0 _- (α_δ_y_δ_x_δβ_α_γ_y_γ_x_γβ_)/2, with 0 ≤ α_γ _≤ C (1 ≤ γ ≤ M) and α_γ_y_γ _= 0, where only the variations that maximize in terms of the α_γ _remain (known as the Wolfe Transformation). In this form the computational task can be greatly simplified. By introducing an expression for the discriminating hyperplane: f_i _= w_β_x_iβ _- b = α_γ_y_γ_x_γβ_x_iβ _- b, the variational solution for L(α) reduces to the following set of relations (known as the Karush-Kuhn-Tucker, or KKT, relations): (i) α_i _= 0 ⇔ y_i_f_i _≥ 1, (ii) 0 < α_i _< C ⇔ y_i_f_i _= 1, and (iii) α_i _= C ⇔ y_i_f_i _≤ 1. When the KKT relations are satisfied for all of the α_γ _(with α_γ_y_γ _= 0 maintained) the solution is achieved. (The constraint α_γ_y_γ _= 0 is satisfied for the initial choice of multipliers by setting the α's associated with the positive training instances to 1/N^(+) ^and the α's associated with the negatives to 1/N^(-)^, where N^(+) ^is the number of positives and N^(-) ^is the number of negatives.) Once the Wolfe transformation is performed it is apparent that the training data (support vectors in particular, KKT class (ii) above) enter into the Lagrangian solely via the inner product x_iβ_x_jβ_. Likewise, the discriminator f_i_, and KKT relations, are also dependent on the data solely via the x_iβ_x_jβ _inner product.

Generalization of the SVM formulation to data-dependent inner products other than x_iβ_x_jβ _are possible and are usually formulated in terms of the family of symmetric positive definite functions (reproducing kernels) satisfying Mercer's conditions [45,46].

The SVM Kernels that are used are based on "regularized" distances or divergences like those used in [8,47], where regularization is achieved by exponentiating the negative of a distance-measure squared (d^2^(x, y)) or a symmetrized divergence measure (D(x, y)), the former if using a geometric heuristic for comparison of feature vectors, the latter if using a distributional heuristic. For the Gaussian Kernel: d^2^(x, y) = Σ_k_(x_k _- y_k_)^2^; for the Absdiff Kernel d^2^(x, y) = (Σ_k_|x_k _- y_k_|)^1/2^; and for the Symmetrized Relative Entropy Kernel D(x, y) = D(x||y) + D(y||x), where D(x||y) is the standard relative entropy.

## Competing interests

The author declares that they have no competing interests.

## Supplementary Material

Additional file 1 Typical pattern of current rise with increase in background electrolyte. Click here for file

Additional file 2 The total resistance vs. MgCl2 is shown, and current through the α-HL channel increases  as electrolyte concentration rises. Click here for file

Additional file 3 The total resistance vs. MgCl2, with MgCl2 concentration increase. Click here for file

Additional file 4 A current blockade signal change is examined with KCl increase. Click here for file

Additional file 5 The 150 feature HMM profile [2] of the data is shown at 1.0, 1.9 and 2.5 M KCl (corresponding to the signal shown in file 4). Click here for file

Additional file 6 An automatically generated plot of spike characteristics for blockade data when DNA hairpins were examined, one radiated and one not. Click here for file
