# Supplementary figures and images for: The α-Hemolysin nanopore transduction detector – single-molecule binding studies and immunological screening of antibodies and aptamers
Source: BMC Bioinformatics. 2007 Nov 1;8(Suppl 7):S9. doi: 10.1186/1471-2105-8-S7-S9 (PMC2099501; doi:10.1186/1471-2105-8-S7-S9)

**2 seconds**


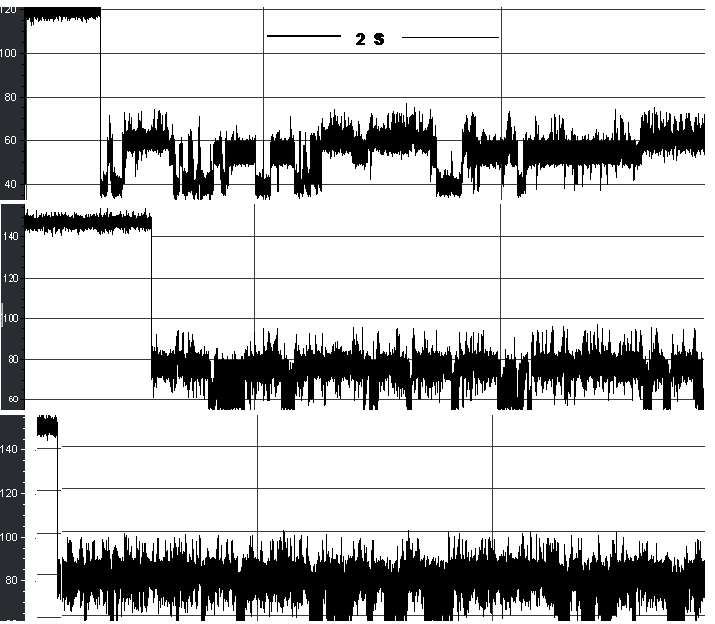


**120**

**155**

**80**

**147**

**60**

**80**

**pA**

**(1)**

**(3)**

**(2)**

**Time**

Supplement: Additional file 4 — A current blockade signal change is examined with KCl increase. [file 1471-2105-8-S7-S9-S4.doc]
