# Supplementary material for: The α-Hemolysin nanopore transduction detector – single-molecule binding studies and immunological screening of antibodies and aptamers
Source: BMC Bioinformatics. 2007 Nov 1;8(Suppl 7):S9. doi: 10.1186/1471-2105-8-S7-S9 (PMC2099501; doi:10.1186/1471-2105-8-S7-S9)
Supplement: Supplementary file 5 [file 1471-2105-8-S7-S9-S6.doc]

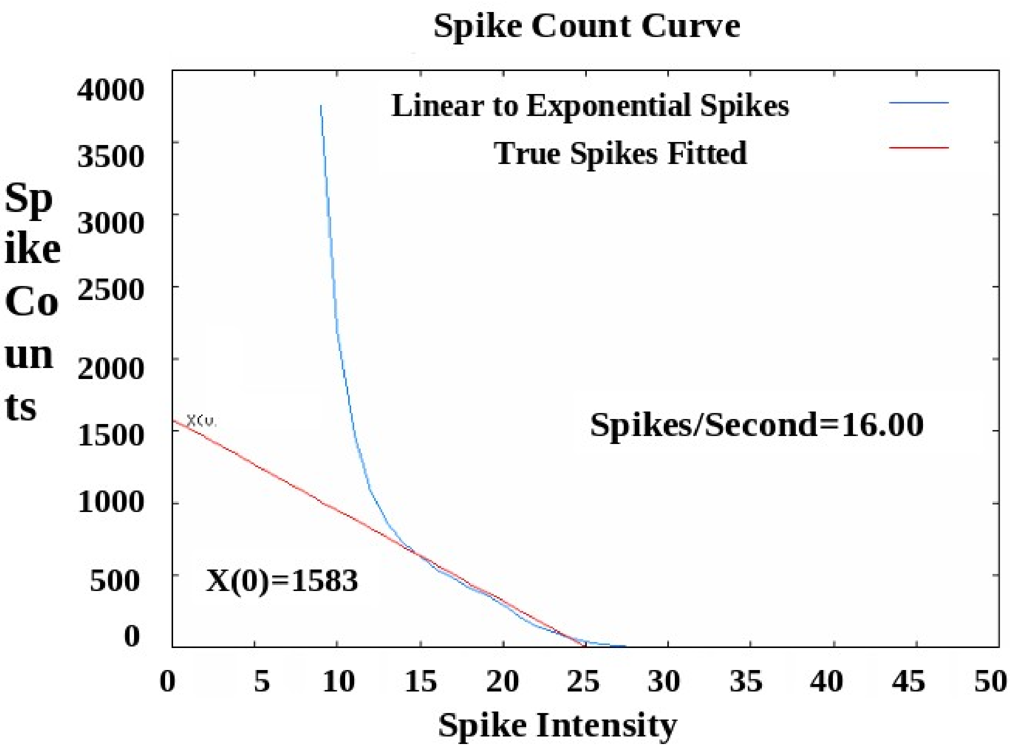

Spike Distribution: Linear to Exponential
